# Intoxication by angel’s trumpet: case report and literature review

**DOI:** 10.1186/1756-0500-7-553

**Published:** 2014-08-20

**Authors:** Yoon Kim, Jinkwon Kim, Ok Joon Kim, Won Chan Kim

**Affiliations:** Department of Neurology, CHA Bundang Medical Center, CHA University, 59 Yatap-ro, Bundang-gu, Seongnam-si, Gyeonggi-do, 463-712 South Korea

**Keywords:** Brugmansia, Anticholinergics, Poisoning

## Abstract

**Background:**

*Brugmansia*, commonly referred to as angel’s trumpet (AT), has been become popular in Korea as an ornamental shrub. However, it is not generally known by the public that this plant contains tropane alkaloids, and that ingestion of AT can lead to anticholinergic poisoning.

**Case presentation:**

A 64-year-old Korean female presented with acute mental changes caused by inadvertent ingestion of the petals of AT flowers used as a garnish in a traditional Korean food (*bibimbop*). She regained her usual level of awareness after 10 hours.

**Conclusion:**

Considering its easy availability, the toxicity of AT should be addressed to prevent accidental and intentional poisoning by this ornamental plant.

## Background

**Figure 1:**
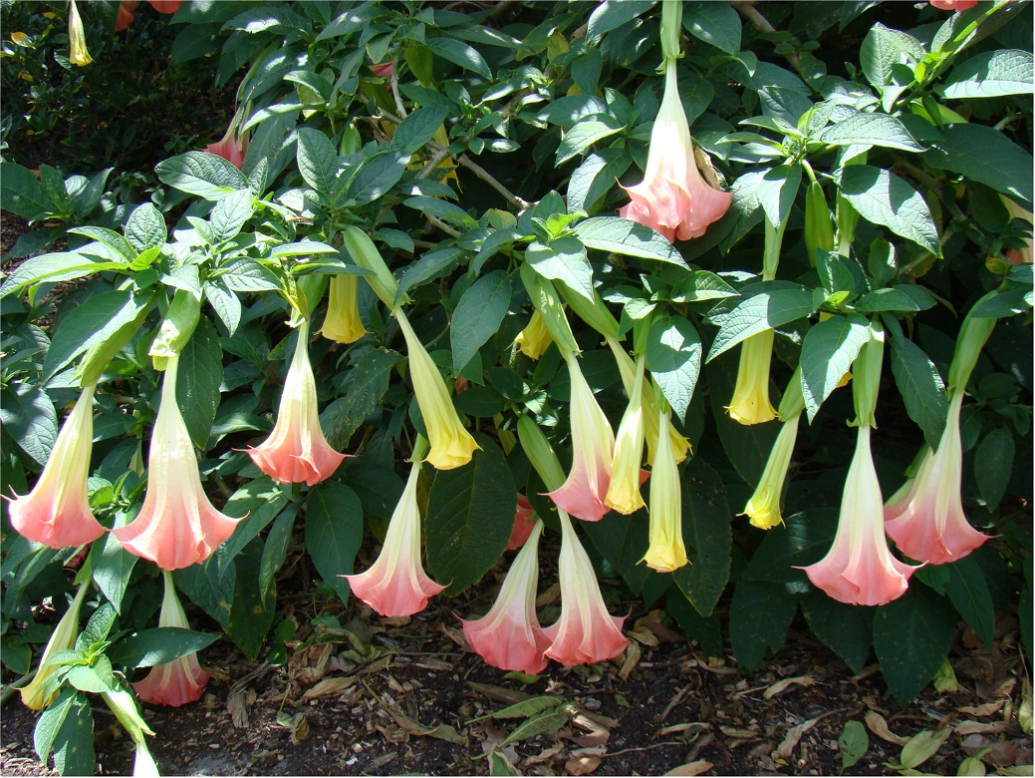
***Brugmansia*** **(angel’s trumpet).** “AngelTrumpet Mounts Asit” by Asit K. Ghosh Thaumaturgist - Own work. Licensed under Creative Commons Attribution-Share Alike 3.0 via Wikimedia Commons - http://commons.wikimedia.org/wiki/File:AngelTrumpet_Mounts_Asit.jpg#mediaviewer/File:AngelTrumpet_Mounts_Asit.jpg.

*Brugmansia*, commonly referred to as angel’s trumpet (AT) (Figure 1), has become popular as an ornamental shrub. Intoxication can occur after drinking AT tea or ingestion of raw AT.

Herein, we report a case of delirium caused by inadvertent intoxication after consumption of *bibimbop* (a traditional Korean dish in which steamed rice, raw/steamed vegetables and barbecued beef are mixed together with a hot pepper paste) garnished with a few petals of AT.

## Case presentation

A 64-year-old Korean female visited the emergency room of our hospital with fluctuating levels of consciousness and dysarthria. Her daughter reported that she noticed dysarthria and incoherent speech when the patient asked for help 20 minutes before they arrived at the hospital.

In the first interview, the patient said she remembered calling her daughter but that she did not remember why. Even though she was alert, she displayed fluctuations in attention, orientation, comprehension and fluency. Otherwise, there were no focal neurologic signs.

Physical examination did not reveal abnormal autonomic features such as tachycardia/bradycardia, mydriasis/miosis, tremor, pallor, dry mouth or sweating. Blood pressure, respiratory rate, and body temperature were all within normal ranges. She did not complain of headache, nausea or vomiting.

Hematologic tests revealed hemoglobin levels, white blood cell count and platelet count to be within normal ranges. Routine blood chemistry displayed an elevated level of low-density lipoprotein-cholesterol (150 mg/dL). Otherwise, there were no abnormal findings that might account for her acute mental change, such as hypoglycemia/hyperglycemia, hyperammonemia or electrolyte imbalance.

Ten hours after ingestion of a meal containing AT, she regained consciousness spontaneously and reported that she had ingested AT flowers in the morning. She said that she added AT to the *bibimbop* her daughter had made for her breakfast, wholly ignorant of its toxicity. Without any intention of self-harm, she stripped a few flowers from the plant she had been growing and added them to the bowl. As soon as she had taken a few spoonfuls of *bibimbop*, she sensed difficulty in speaking. Panicked, she immediately called her daughter for help. Twenty-four hours after ingestion of AT, she had only partial recollection of the 10 hours immediately after the intoxication. Otherwise, she was not different from her usual self.

## Discussion

AT, originally native to South America, is a widely cultivated ornamental shrub in Western Europe, Southeastern United States, Australia and Asia [1]. It has been naturalized in Korea as well. The plant stands 8–15 feet in height. Its leaves are shaped like lancets or eggs. The trumpet-shaped flowers come primarily in white, yellow or red colors, and measure 10–12 inches in diameter. These fragrant flowers hang straight down, endowing them with a unique appearance (Figure 1). The plant has been distributed widely and cultivated as a garden plant because of its low maintenance needs and beauty. However, it is not well known by the general public that AT is poisonous. Hence, poisoning by ingestion of AT is not uncommon.

AT contains tropane alkaloids such as scopolamine, hyoscyamine and atropine. They are embedded in virtually all parts of the plant. Any part of the plant could contain toxins but most are in the roots and seeds [1]. There is considerable variation in the concentration of these substances depending on the plant part tested, the season, the stage of maturation, and the state of hydration [2, 3].

The alkaloids contained in the plant (most notably scopolamine) cause post-synaptic competitive inhibition of cholinergic muscarinic receptors (in central and peripheral regions), resulting in the classic picture of anticholinergic poisoning seen in AT intoxication. Symptoms develop rapidly, sometimes as early as 5–10 minutes after ingestion [2]. In AT intoxication, patients initially experience salivation and sweating, followed by a dry mouth, mydriasis, and loss of accommodation and tachycardia. As the dose increases, urinary retention and hyperthermia ensue. At this point, confusion, agitation and anxiety can be witnessed as being due to acute anticholinergic poisoning by the plant. Neuropsychologic assessment of the patient can reveal impairment of orientation, affective lability, incoherent thoughts, flight of ideas, tangential thinking, and auditory or visual hallucinations [1]. Amnesia with regard to the events immediately after ingestion along with mydriasis may last for days even after remission of other neuropsychiatric symptoms. At even higher doses, convulsions, flaccid paralysis and delirium can occur [4]. If not recognized and treated promptly, severe intoxications may lead to death. In severe cases, the cholinesterase inhibitor physostigmine can be given *via* the intravenous route as an antidote. Gastric lavage and administration of activated charcoal are recommended ≤48 hours after ingestion [1]. Each blossom contains ≈ 0.65 mg scopolamine and 0.3 mg atropine. Fatalities have been reported at an atropine dose of 10 mg [2]. As such, ingestion with as few as 10 flowers can be not only toxic but also fatal.

Easy availability of AT has been suggested to be one reason behind its frequent abuse in western countries [1]. It was not until the 1970s in the USA when reports of intoxication by AT and Jimson weed increased as adolescents were poisoned inadvertently while consuming them for recreational purposes. In 1994 alone, in the state of Florida, USA, 85 cases of intentional ingestion of AT were reported [2]. Among the abusers of this plant, some chose to eat the blossoms while others smoked the leaves. The most popular method of ingestion has been steeping the blossoms and seeds in water to obtain a tea. This preparation is sometimes combined with alcohol or cannabis in the hope of amplifying its potential as a hallucinogen [2].

## Conclusions

This case is unique in that AT was ingested as an ingredient of a traditional Korean dish. This poisonous shrub, having been grown for ornamental purposes, has gained popularity over recent years. Considering the fact that one can purchase it from virtually any florist without much difficulty, and that the number of adolescent recreational drug users is increasing, AT could be misused in the near future. The flowers of AT are occasionally used to garnish foods, so raising the awareness of the toxicities of this plant to the general public is important.

## Consent

Written informed consent was obtained from the patient for publication of this Case report and any accompanying images. A copy of the written consent is available for review by the Editor-in-Chief of this journal.

## Abbreviation

AT: Angel’s trumpet.
